# Multi-heterodyne two dimensional coherent spectroscopy using frequency combs

**DOI:** 10.1038/s41598-017-14537-z

**Published:** 2017-10-25

**Authors:** Bachana Lomsadze, Steven T. Cundiff

**Affiliations:** 1 Department of Physics, University of Michigan, Ann Arbor, Michigan 48109 USA; 2 JILA, University of Colorado & National Institute of Standards and Technology, Boulder, Colorado, 80309 USA

## Abstract

Optical multi-dimensional coherent spectroscopy is a powerful technique for studying the structure, properties and ultrafast dynamics of atoms, molecules, semiconductor materials and complex systems. Current implementations of multi-dimensional coherent spectroscopy have long acquisition times and/or limited spectral resolution. In addition, most of the techniques utilize complex geometries or phase cycling schemes to isolate non-linear signals. We demonstrate a novel approach of using frequency combs to perform rapid, high resolution and background free multi-dimensional coherent spectroscopy of semiconductor materials. Our approach is inspired by dual-comb spectroscopy, which has been proven to be a versatile tool for obtaining one dimensional absorption spectra with high resolution in a short acquisition time. We demonstrate the method using a GaAs multi-quantum well sample.

## Introduction

Rapid and precise measurements of light-matter interactions are crucial in understanding the complex properties of materials^1–8^. Since the development of frequency combs (typically mode-locked lasers), the technique known as dual comb spectroscopy (DCS)^9,10^ has attracted substantial attention as a revolutionary approach to optical spectroscopy. DCS is analogous to Fourier Transform Infrared (FTIR) spectroscopy but does not require moving optical elements, which limit the acquisition speed. DCS is a powerful technique that combines high spectral resolution, high sensitivity, broad spectral coverage and fast acquisition speeds. Because of these qualities, DCS is being developed for use in many fields, from atomic and molecular spectroscopy, to precision metrology, to light detection and ranging (LIDAR) and even atmospheric monitoring^11–15^. In DCS, one frequency comb interrogates the sample, and the linear response is sampled in time with a LO comb with a slightly different repetition rate. The resulting interferogram is captured by a single photodetector. In the frequency domain, this arrangement produces a Radio Frequency (RF) comb spectrum that results from these two optical combs beating against each other on a single photodetector. With the development of micro-resonator combs^16^ DCS is becoming transportable as well. However, DCS suffers from the limitations inherent in one dimensional techniques arising from inhomogeneous broadening and the inability to discern if resonances are coupled.

Optical multi-dimensional coherent spectroscopy (MDCS) has been proven to be a very powerful tool for addressing these limitations. The concept of MDCS originated in Nuclear Magnetic Resonance^17^ where it is widely used today. Optical MDCS is an advanced non-linear technique that uses a sequence of laser pulses (typically three) incident to the sample and detects the Four-Wave-Mixing (FWM) signal generated by the sample as a function of the time delay(s) between the incident pulses. A multi-dimensional spectrum is then constructed by Fourier transforming the signal with respect to the emission time and the delays between the pulses. Optical MDCS has many advantages including 1) unfolding congested one dimensional spectra and isolating different spectral contributions, 2) identifying couplings and interactions between excited states, 3) separating homogeneous and inhomogeneous linewidths, and 4) providing important insights about many-body physics. However, high resolution MDCS techniques (implemented using long delay stages to probe narrow resonances such as atoms, molecules, Nitrogen vacancy centers, self-assembled quantum dots etc.) have long acquisition times (tens of minutes to hours), whereas rapid MDCS techniques provide much lower spectral resolution^18–21^. Spectral resolution limitation is either due to time delays (between pulses) achievable using acousto-optic pulse shapers, spatial spectral interferometry or due to the spectrometer resolution. In addition, the experimental apparatus is bulky and uses complex geometries and phase cycling schemes to suppress background signals.

Recently we have performed a marriage between MDCS and DCS, which we call M-DSC^2^, that allows us to perform multi-dimensional coherent spectroscopy that is simultaneously rapid, background free and high resolution^22^. The method was applied to atomic samples and here, we extend its applications for semiconductor materials. DCS technique has previously been applied to coherent anti-stokes Raman spectroscopy and time resolved pump-probe spectroscopy^23,24^ to study molecules and semiconductor materials. M-DCS^2^ extends its applications for multi-dimensional coherent spectroscopy.

## Method

**Figure 1:**
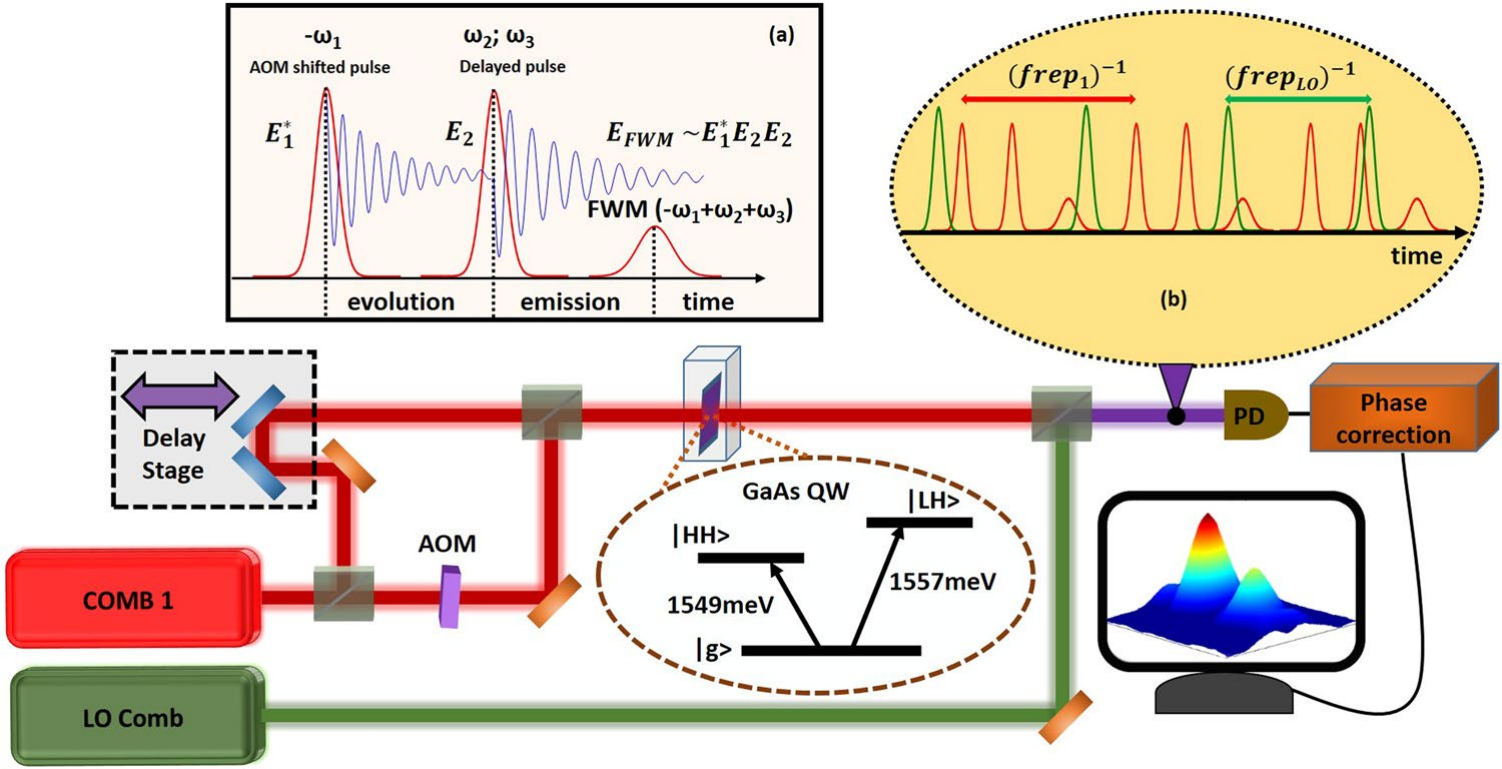
Experimental setup: Train of pulses from comb 1 is split into two parts. One part is frequency shifted using an acousto-optical modulator (AOM) and combined with the other part. The combined beam interact with the GaAs quantum well sample. The sample represents the “V” system with coupled (via the ground state) Heavy Hole (HH) and Light Hole (LH) resonances. Inset (**a)** shows the generation of a Four Wave mixing (FWM) signal in photon echo scheme. Blue traces correspond to evolution and emission of the coherences created by the sequence of pulses. Generated FWM signal along with the incident pulses are combined with the train of the LO comb having a slightly different repetition rate. Inset **(b)** shows the LO comb pulses (green) sweeping through the excitation and FWM pulses (red). The combined beams are interfered on a photodetector (PD). Before digitizing, the phase fluctuations are measured and corrected in real time. The evolution of the FWM signal is measured by sweeping the delay stage.

The concept of comb based MDCS is schematically shown in Fig. 1 (please the supplementary material for details). Two combs with different offset frequencies, produced from a single source comb using an Acousto-Optical Modulator (AOM), generate a FWM signal in a collinear geometry. Inset (a) shows the generation of a FWM signal by a pair of pulses (delayed pulse interacts twice (supplementary material) in the photon echo sequence^25^. The emitted FWM comb is then spectrally separated in the RF domain after heterodyne detection with the Local Oscillator (LO) comb^26^ that has a slightly different repetition rate (f_rep_1_ = 93.544290 MHz and f_rep_LO_ = 93.544290 MHz + 220 Hz locked to a direct digital synthesizer). The repetition rate difference allows the LO delay to be scanned rapidly over a long range (Fig. 1 (inset b)) and is a distinction from AOM based approaches that only use a single laser^27^. The delay between the excitation pulses is then varied using a retroreflector mounted on a mechanical translation stage to generate the second dimension for the two dimensional spectrum. The phase fluctuations due to fluctuations in offset frequency, optical path length and/or repetition frequency are measured and subtracted in real time^26^.

For this proof of concept experiment, we used a sample containing 10 layers of 10 nm thick GaAs quantum wells (QW) separated by the same thickness Al_0.3_Ga_0.7_As barriers cooled down to 7 K. The optical bandwidth of the beams are filtered such that they excite both the Heavy Hole (HH) and the Light Hole (LH) excitonic resonances (level diagram shown in Fig. 1). The optical bandwidth that can unambiguously be mapped to the RF domain strongly depends on how tightly the lasers are phase locked. Our phase locking loops and cancelation scheme^26^ allowed us to obtain a comb resolution in a limited optical range. For this proof of concept experiment we applied real time phase correction in the optical region covering the HH resonance and hence an optical bandpass filter (3 nm full width at half maximum) covering only the HH resonance was inserted before the photodetector.

## Results and Discussion

**Figure 2:**
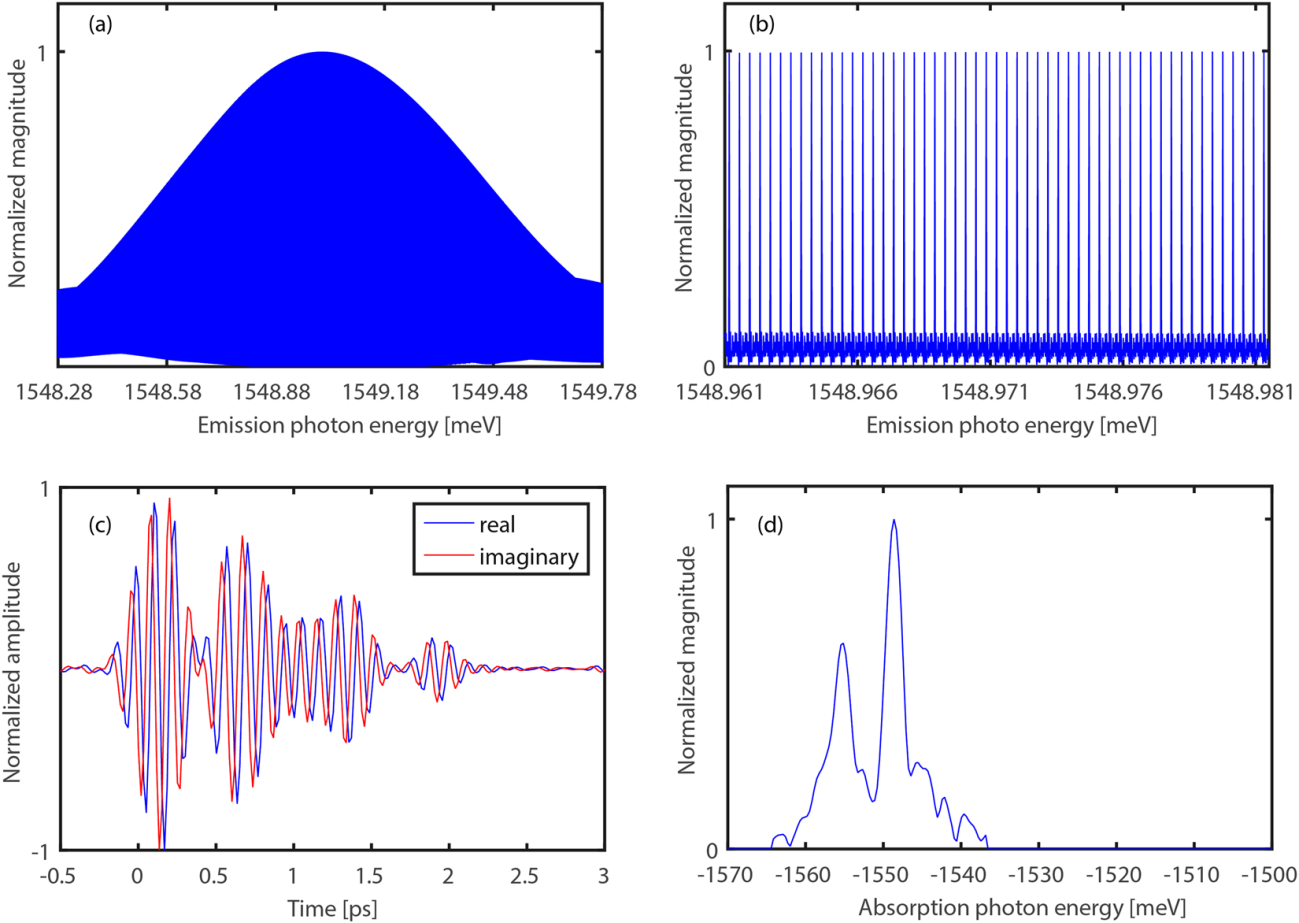
Experimental results. (**a)** Spectrum of the FWM signal at zero delay, **(b)** Portion of (A) showing the comb structure. **(c)** Evolution of real (blue) and imaginary (red) parts of single comb’s electric field as a function of stage delay. **(d)** Fourier transform of the beat pattern of (C) showing HH (−1549 meV) and LH (1557 meV) absorption energies.

Figure 2(a) shows the spectrum of the FWM signal at zero delay remapped to the optical domain. This spectrum corresponds to the Fourier transform of the emitted FWM signal with respect to the emission time (Fig. 1 inset (a)). A portion of the spectrum on an expanded scale is shown in Fig. 2(b), the comb structure is clear. This spectrum was obtained in 100 ms and the width of a single comb tooth is a few MHz (~10 neV). In traditional time-domain spectroscopy, similar resolution in the same acquisition time would require a >30 m long delay stage moving with nearly the speed of sound. Currently available commercial FTIR spectrometers have the maximum path length difference up to 10 m and can provide the resolution of 30 MHz.

To generate a two dimensional spectrum, we scanned the delay between excitation pulses with 16.7 fs steps and recorded the FWM RF signal at each delay. Figure 2(c) shows the evolution of the real and imaginary parts of a single comb line’s (the middle comb tooth on Fig. 2(b)) electric field as a function of the excitation delay. It displays a beat pattern that corresponds to the interference of two FWM signals emitted at the HH excitonic resonance frequency that have different absorption (evolution) frequencies, specifically the HH and LH excitonic resonances. The electric field of the signal is

1 $$\begin{array}{c}{{\boldsymbol{E}}}_{{\boldsymbol{sig}}} \sim {{\boldsymbol{E}}}_{1}^{\ast }({{\boldsymbol{\omega }}}_{{\boldsymbol{lh}}}){{\boldsymbol{E}}}_{2}({{\boldsymbol{\omega }}}_{{\boldsymbol{hh}}}){{\boldsymbol{E}}}_{2}({{\boldsymbol{\omega }}}_{{\boldsymbol{lh}}}){{\boldsymbol{e}}}^{-{\boldsymbol{i}}{{\boldsymbol{\omega }}}_{{\boldsymbol{lh}}}{\boldsymbol{\tau }}}+{{\boldsymbol{E}}}_{1}^{\ast }({{\boldsymbol{\omega }}}_{{\boldsymbol{lh}}}){{\boldsymbol{E}}}_{2}({{\boldsymbol{\omega }}}_{{\boldsymbol{lh}}}){{\boldsymbol{E}}}_{2}({{\boldsymbol{\omega }}}_{{\boldsymbol{hh}}}){{\boldsymbol{e}}}^{-{\boldsymbol{i}}{{\boldsymbol{\omega }}}_{{\boldsymbol{lh}}}{\boldsymbol{\tau }}}+\\ 2{{\boldsymbol{E}}}_{1}^{\ast }({{\boldsymbol{\omega }}}_{{\boldsymbol{hh}}}){{\boldsymbol{E}}}_{2}({{\boldsymbol{\omega }}}_{{\boldsymbol{hh}}}){{\boldsymbol{E}}}_{2}({{\boldsymbol{\omega }}}_{{\boldsymbol{hh}}}){{\boldsymbol{e}}}^{-{\boldsymbol{i}}{{\boldsymbol{\omega }}}_{{\boldsymbol{hh}}}{\boldsymbol{\tau }}}\end{array}$$

where ${{\boldsymbol{\omega }}}_{{\boldsymbol{hh}}}$ and ${{\boldsymbol{\omega }}}_{{\boldsymbol{lh}}}$ correspond to HH and LH frequencies and ${\boldsymbol{\tau }}$ is the evolution time (Supplementary text). The Fourier transform of the beat signal with respect to the evolution time is shown in Fig. 2(d). The peaks correspond to HH and LH absorption energies. The negative sign on the absorption energy axis is due to the negative phase evolution during the evolution period (Fig. 1 inset (a)).

**Figure 3:**
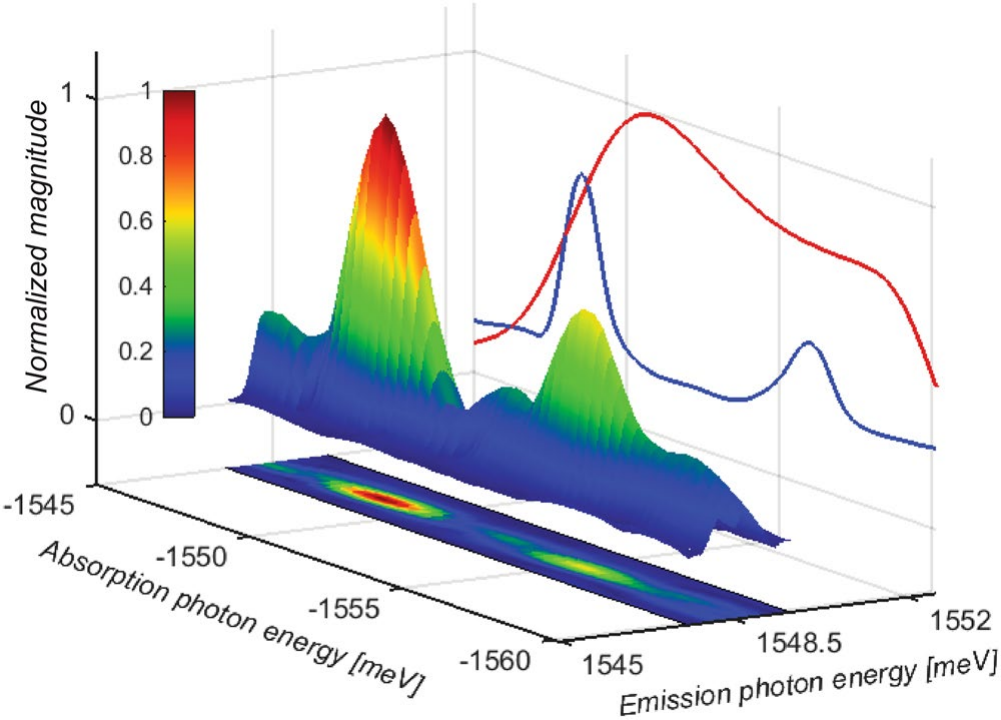
Two dimensional energy spectrum measured at HH emission energy (1549 meV). Bottom plane corresponds to the projection. The spectrum correlates emission energy to absorption energy. The peak at (−1549, 1549) corresponds to absorption and emission at HH energy. The peak at (−1549, 1556) corresponds to absorption at LH energy and emission at HH energy. The side plane shows the measured linear absorption spectrum of the sample (Blue) and laser spectrum (Red) before the sample.

Figure 3 shows the two dimensional coherent spectrum obtained by Fourier transforming all comb lines in the FWM spectrum with respect to the evolution time. On the bottom plane, the same data is shown using a contour plot. On the side plane, the measured linear absorption spectrum (blue) and excitation laser spectrum (red) are shown. The two-dimensional spectrum covers a rectangular region in frequency space because of the restricted the bandwidth over which we get comb resolution, improving to the repetition rate lock or implementing adaptive sampling^28^ will increase the bandwidth.

The two-dimensional spectrum shows two peaks. The peak at −1549 meV absorption photon energy and 1549 meV emission photon energy corresponds to absorption and emission at the HH exciton energy whereas the coupling between the HH and LH exciton resonances results in the peak at −1557 meV absorption photon energy and 1549 emission photon energy. Similar coupling information can be obtained if the emission of the FWM signal is detected at LH energy (1557 meV). In either case, if HH and LH resonances represented 2 uncoupled resonances, then the coupling peak would be absent. The coupling information, in addition to the rapid acquisition and high resolution associated with frequency combs, can potentially be used as an efficient way to separate different species in a sample containing a mixture, which cannot be achieved using only one dimensional high resolution energy spectra.

In addition to the high resolution, our method provides a route to very short acquisition times, which is critical in many applications. In this experiment, the full two dimensional spectrum can be obtained under 3 minutes, which can be improved by optimizing the stage performance. A more dramatic speed-up could be realized by using three frequency combs with different repetition rates to improve the resolution and acquisition speed in the evolution time as well. Three combs would allow the same spectrum with comb resolution in both energy axes to be obtained in a few seconds (Supplementary text).

## Electronic supplementary material

Supplementary Information
