# Supplementary material for: Multi-heterodyne two dimensional coherent spectroscopy using frequency combs
Source: Sci Rep. 2017 Oct 25;7:14018. doi: 10.1038/s41598-017-14537-z (PMC5656649; doi:10.1038/s41598-017-14537-z)
Supplement: Supplementary file 1 — Supplementary Information [file 41598_2017_14537_MOESM1_ESM.docx]

Supplementary Materials

**Multi-heterodyne two dimensional coherent spectroscopy using frequency combs**

**Bachana Lomsadze^1,2^ and Steven T. Cundiff ^1,2,*^**

**^1^Department of Physics, University of Michigan, Ann Arbor, Michigan 48109, USA**

**^2^JILA, University of Colorado & National Institute of Standards and Technology, Boulder, Colorado 80309, USA**

***Correspondence to**: **cundiff@umich.edu**

**This file includes:**

Supplementary Text

Supplementary Figure 1

Supplementary Figure 2

Supplementary Figure 3

Supplementary Text

Supplementary Figure 1 shows a schematic diagram of the experimental setup. The details can be found elsewhere in Ref 27*.* We used two Kerr-lens mode-locked Ti:Sapphire lasers centered at 800 nm. The repetition frequencies for Comb 1 and LO comb (f_rep_1_=93.544290 MHz and f_rep_LO_=93.544290MHz+220 Hz) were phase locked to a direct digital synthesizer, but the comb offset frequencies were not actively stabilized.

Using a half wave plate and a polarizing beam splitter (PBS 1) the output of comb 1 was split into 2 parts. One part was frequency shifted by 80 MHz using an Accousto-optical modulator and combined with the other part on PBS 2. The delay between the two pulse trains was adjusted and controlled using a delay stage. The combined beams were projected on the same linear polarization state using a polarizer and interacted with the GaAS quantum well sample that was cooled down to 7 K. The optical spectra for the excitation beams covered both the Heavy Hole (HH) and the Light Hole (LH) excitonic resonances. Average powers for beams travelling through path 1 and path 2 were 1.3 and 2.6 mW respectively and were focused to 30 um spot on the sample. The four-wave-mixing FWM signals emitted by the sample along with the incident beams were combined with the LO comb on PBS 3. Half wave plates were adjusted such that only a small fraction of the light from each beam was sent to PBS 4 to monitor the optical phase fluctuations whereas most of the light was sent to Det 1 photodetector to obtain a RF FWM spectrum. Before interfering, the beams were spectrally filtered using an optical line filter to detect the light at HH resonance (the reason explained in the main text).

Generation of FWM signal in photon echo excitation sequence.

Supplementary figure 2 shows the generation of the FWM signal both in the time and frequency domains. In the time domain the FWM signal is generated by the sequence of pulses. The first pulse (phase conjugated pulse) excites the coherence between the ground and excited states (blue trace on the figure shows the evolution of the coherence in time), the second pulse converts this coherence into the excited state population and then it converts back to the coherent superposition state that radiates the FWM signal (blue trace on the figure). For clarity we separated the second and third interactions (on suppl. figure 2) to show the conversion into the population state but in the experiment these interactions happen at the same time (second pulse interacts twice).

In the frequency domain (right figure) the blue lines correspond to the AOM shifted comb lines, black lines correspond to the original comb lines and the red and magenta lines correspond to the generated FWM signals $E_{1}^{*}E_{2}E_{2}$ and $E_{2}^{*}E_{1}E_{1}$ (Ref. 27). The green lines correspond to the LO teeth. Det 1 on Supplementary Figure 1 measures the interference between blue, black, red and magenta lines with the green lines which results into producing a multi-heterodyne beat signals in the RF domain (supplementary figure 2) where the linear and FWM signals are spectrally separated. In the experiment we isolate only the red portion of the FWM RF signal using a RF bandpass filter.

We would like to note that both FWM signals exist only at zero time delay between pulses but due to causality only one survives for the finite delay. One can measure the other signal by moving the delay stage in the opposite direction.

Interference of FWM signals emitted at HH resonance.

Supplementary figure 3 shows the double sided Feynman diagrams that correspond to the equation shown in the main text. (a) and (b) diagrams correspond to the generation of the FWM signals that have the same HH absorption (evolution) and emission resonance frequencies (third term in the equation). (c) and (d) diagrams correspond to the FWM signals emitted at the HH resonance frequency that evolved with LH resonance frequency (first and second terms in the equation). For clarity we separated the second and third interactions (middle dashed lines) but in the experiment the interactions happen at the same time (second pulse interacts twice).

Phase correction

To obtain a multi-dimensional spectrum with comb resolution the phase fluctuations due to the lasers’ optical frequency and path fluctuations have to be monitored (details described in reference 27). We used 2 Continuous Wave (CW) lasers to track all (relevant) phase fluctuations. CW 1 laser, tuned near HH resonance, was used to measure the optical phase fluctuations of LO comb and comb 1 (caused by the offset and residual repetition frequency fluctuations) on Det 3 and Det 4 respectively. It also served as a marker to frequency remap from RF to optical domain. CW 2 laser, tuned at 784 nm, was used to track the path fluctuations between path 1 and path 2 on Det 2. It also served as a reference for the FWM signal phase evolution during the evolution period (scanning the stage). We would like to note that the fluctuations can be tracked using only CW 1 laser (tuned near HH resonance) but we used the second laser to avoid the detection of the evolution phases near zero.

The signals from Det2, Det 3 and Det 4 were used to generate the correction signal and mixed with the RF FWM signal from Det 1(Ref S1). The phase fluctuation free signal was digitized using a fast (250 MHz sampling rate) data acquisition board.

We would like to note that our phase cancelation scheme treats repetition frequency fluctuations as offset frequency fluctuations and degrades for the teeth that are far away from the CW laser. This can be improved by tight locking the repetition frequencies or by implementing adaptive sampling (ref 30 ) that uses two CW lasers that decouples the offset and repetition frequency fluctuations. This would also improve the SNR and remove side lobes on our 2d spectrum. In addition choosing the second CW laser (that monitors the relative path length fluctuations between the excitation pulses) with wavelength closer to HH or LH transitions (~ 800, 796 nm) would improve SNR as well. SNR will also be improved by implementing third frequency comb due to dramatic speed up in acquisition time. For semiconductor materials (that have relatively short lived excited states) high repetition frequency lasers (>1GHz) are preferred to reduce the duty cycle and increase the number of interferograms (to perform averaging for better SNR).


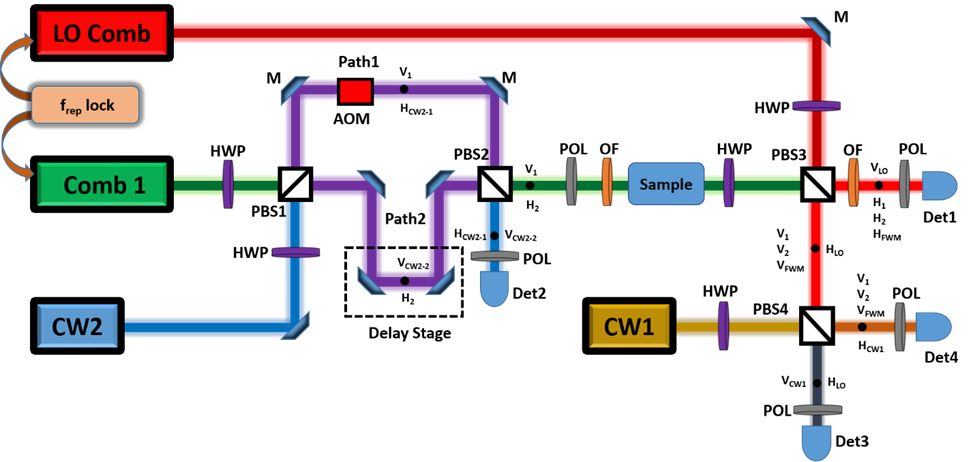


**Supplementary Figure 1** **| Experimental setup**: LO- local Oscillator, CW-continuous wave laser, HWP-Half Wave Plate, PBS-polarizing Beam Splitter, M-Mirror, AOM –Accousto Optical Modulator, POL-Polarizer, OF-Optical Filter, BPF-Band Pass Filter (RF), Det-photodetector. H-Horizontal and V- Vertical Linear polarization states of the beams. Subscripts 1 and 2 indicate the path of the beams.


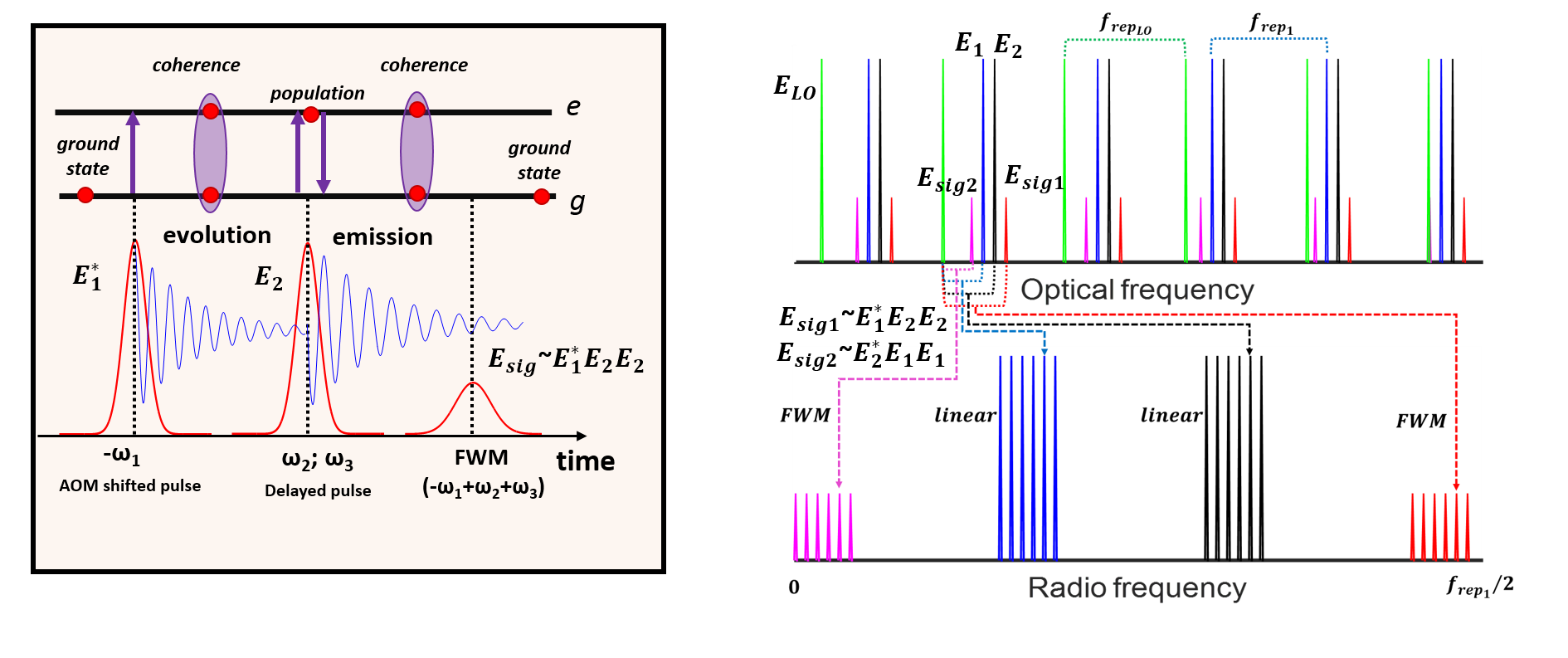


**Supplementary Figure 2** **| FWM generation**: Left-time domain picture. Right-frequency domain picture. Please see the supplementary text for details.


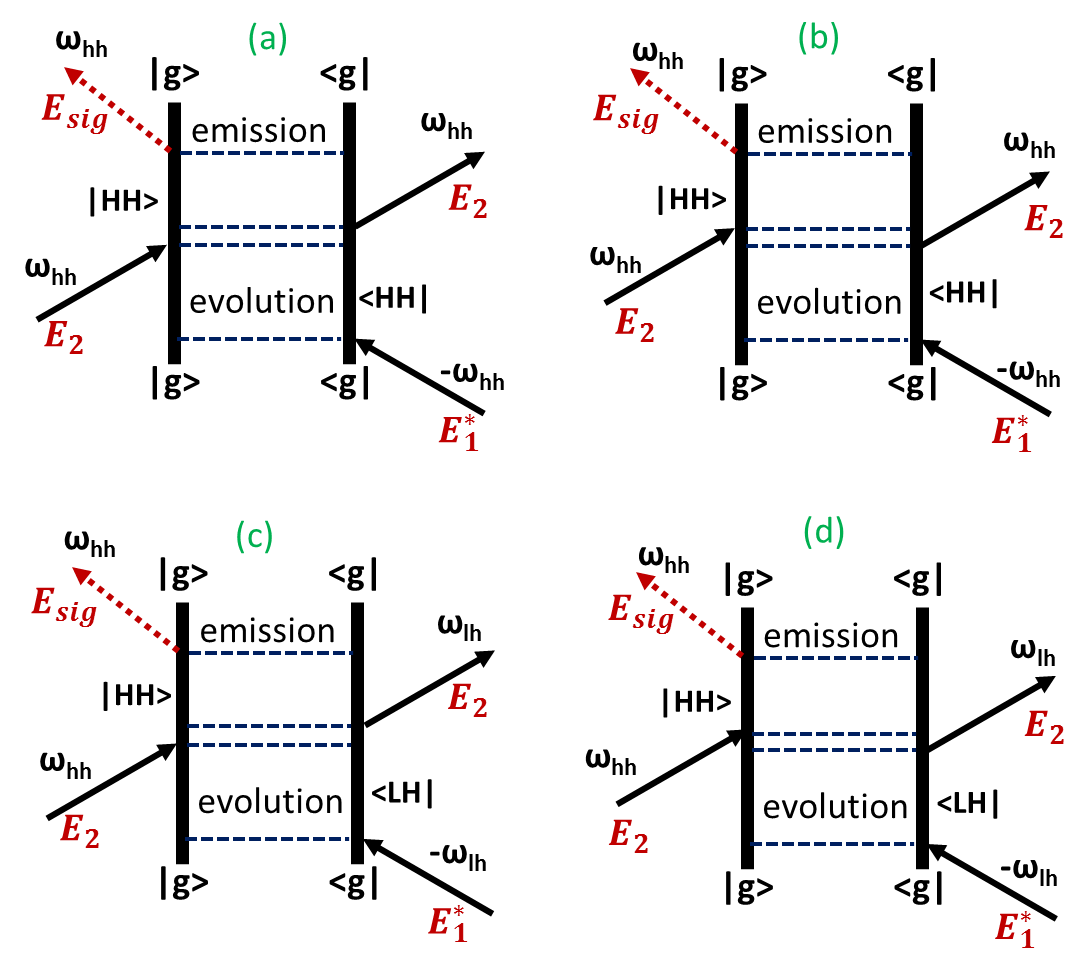


**Supplementary Figure 3** **| Double sided Feynman diagrams**: (a) and (b) correspond to absorption and emission at HH resonance frequency. (c) and (d) correspond to absorption at LH and emission at HH resonance frequencies.
